# The effect of the chemical chaperone 4-phenylbutyrate on secretion and activity of the p.Q160R missense variant of coagulation factor FVII

**DOI:** 10.1186/s13578-019-0333-8

**Published:** 2019-08-27

**Authors:** Elisabeth Andersen, Maria Eugenia Chollet, Marcello Baroni, Mirko Pinotti, Francesco Bernardi, Ellen Skarpen, Per Morten Sandset, Grethe Skretting

**Affiliations:** 1 Department of Haematology, Oslo University Hospital, Oslo, Norway; 2 Research Institute of Internal Medicine, Oslo University Hospital, Oslo, Norway; 3 Institute of Clinical Medicine, University of Oslo, Oslo, Norway; 4 Department of Life Sciences and Biotechnology, and LTTA Centre, University of Ferrara, Oslo, Norway; 5 Core Facility for Advanced Light Microscopy, Institute for Cancer Research, Oslo University Hospital, Ferrara, Italy

**Keywords:** Factor VII deficiency, Chemical chaperones, Mutations, Protein misfolding, Endoplasmic reticulum, Trafficking

## Abstract

**Background:**

Congenital coagulation factor (F) VII deficiency is a rare bleeding disorder caused by mutations in the *F7* gene. The missense factor FVII variant p.Q160R is the disease-causing mutation in all Norwegian FVII deficient patients and results in reduced biological activity and antigen levels of FVII in patient plasma. Previous in vitro studies on this variant demonstrated impaired intracellular trafficking and reduced secretion, possibly due to protein misfolding. The aim of the study was therefore to assess the impact of chemical chaperones on cellular processing and secretion of this variant using a cell model based on overexpression of the recombinant protein.

**Results:**

Through screening of compounds, we identified 4-phenylbutyrate (4-PBA) to increase the secretion of recombinant (r) FVII-160R by ~ 2.5-fold. Additionally, treatment with 4-PBA resulted in a modest increase in specific biological activity. Intracellular localization studies revealed that upon treatment with 4-PBA, rFVII-160R was secreted through Golgi and Golgi reassembly-stacking protein (GRASP)-structures.

**Conclusions:**

The present study demonstrates that the chemical chaperone 4-PBA, restores intracellular trafficking and increases the secretion of a missense FVII variant with functional properties in the extrinsic coagulation pathway.

## Background

Coagulation factor (F) VII is a serine protease that is synthesized in the liver and secreted into the blood where it circulates at a concentration of about 0.5 µg/ml (10 nM) [1]. The FVII protein contains an amino-terminal γ-carboxyglutamic acid domain followed by two epidermal growth factor (EGF)-like domains, and a carboxyl (C)-terminal protease domain [2]. The majority of FVII circulates in its single-chain zymogen form and, after endothelial rupture, it interacts with the transmembrane receptor, tissue factor (TF), and is subsequently converted into the two-chain active (A) form [3]. The formation of the TF-FVIIa (extrinsic tenase) complex is considered the key initiator of blood coagulation [4], activating factor X (FX) and also small amounts of factor IX (FIX) [4].

Congenital factor FVII deficiency is a rare, autosomal recessive bleeding disorder caused by mutations in the *F7* gene resulting in reduced activity (FVII:C) and/or antigen (FVII:Ag) levels in plasma [5]. It is a rare bleeding disorder (1:500,000, [5–7]) with a higher frequency in Norway (1:60,000, unpublished observations). The missense FVII variant p.Q160R (legacy name Q100R) in the second EGF-domain involves an A to G transition of nucleotide 7834, altering a glutamine to an arginine at codon 160. This variant, reported in the FVII variant database [8], is present in all the Norwegian FVII deficient patients and is associated with a bleeding phenotype due to reduced FVII antigen and activity in plasma [9]. Recombinant (r) FVII Q160R (FVII-160R) has been shown to be poorly secreted and, in the presence of relipidated TF, the activated rFVII-160R showed less than 5% of the ability of wild type FVIIa to activate factor X [10].

Our previous in vitro studies on the p.Q160R variant demonstrated impaired secretion due to retention in the endoplasmic reticulum (ER) and binding to ER chaperones [11, 12], possibly as a result of protein misfolding. Chemical chaperones are small compounds that can stabilize misfolded proteins and improve the protein-folding capacity of cells and thereby increase protein secretion [13, 14]. Despite their therapeutic potential, only a few studies have been performed on their effect on mutated coagulation proteins [15–17], and no studies have so far been performed on FVII. In the present study, we therefore examined the potential effects of chemical chaperones on trafficking and secretion of the FVII variant p.Q160R. We found that treatment with 4-phenylbutyrate (4-PBA) increased the biosynthesis and secretion of recombinant (r) FVII-160R produced in Chinese hamster ovary (CHO)-K1 cells and additionally slightly enhanced its biological activity. Investigation of the subcellular localization revealed that 4-PBA could correct the aberrant intracellular trafficking of rFVII-160R, which was transported through Golgi- and Golgi reassembly-stacking protein (GRASP)-structures upon treatment.

## Results

### Treatment with 4-PBA increases biosynthesis and secretion of rFVII-160R

**Fig. 1:**
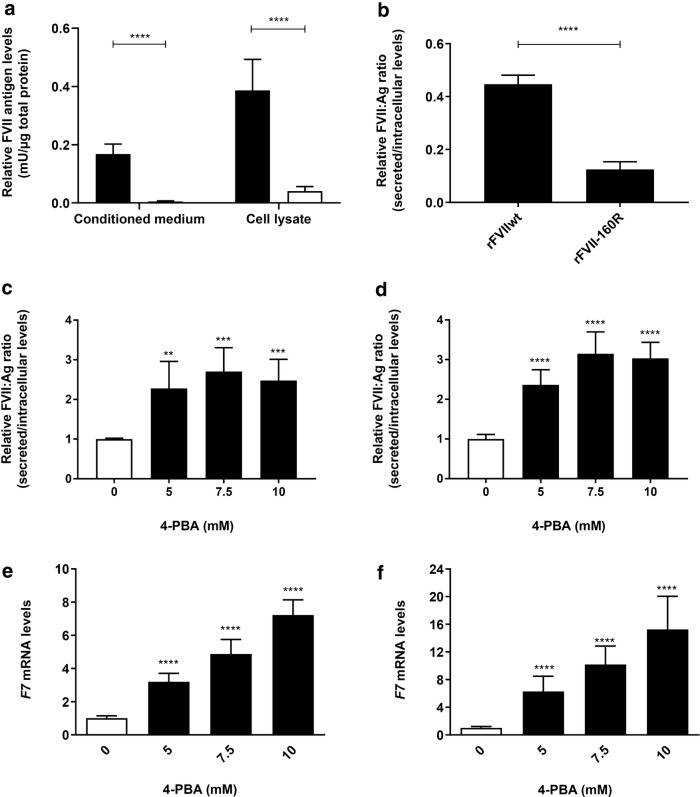
Effect of 4-PBA on secretion of rFVII-160R and rFVIIwt. Cells stably expressing rFVII-160R or rFVIIwt were treated with 0 (untreated), 5, 7.5 or 10 mM 4-PBA for 48 h. **a**–**d** FVII:Ag levels in conditioned medium (secreted) or cell lysates (intracellular) were examined by ELISA. To adjust for variations in cell number, the FVII:Ag levels were adjusted to the corresponding total protein content in the respective wells. **a** FVII:Ag levels in conditioned medium or cell lysates from untreated cells expressing rFVIIwt (black bars) or rFVII-160R (white bars). Values are reported as mean ± SD from at least three independent experiments performed in duplicates (unpaired t-test, ****p ≤ 0.0001). **b** Ratio between secreted/intracellular FVII:Ag levels in untreated cells. Values are reported as mean ± SD from at least three independent experiments performed in duplicates (unpaired t-test, ****p ≤ 0.0001). **c**, **d** FVII:Ag levels in the conditioned medium or cell lysates from cells expressing rFVII-160R (**c**) or rFVIIwt (**d**), treated with 4-PBA were examined by ELISA, and a ratio between secreted/intracellular FVII:Ag was calculated. Values are expressed relative to untreated cells and are reported as mean ± SD from at least three independent experiments performed in duplicates (one-way ANOVA, ****p ≤ 0.0001, ***p ≤ 0.001, **p ≤ 0.01). *F7* mRNA levels from cells expressing rFVII-160R (**e**) or rFVIIwt (**f**), treated with 4-PBA were analysed by Taqman qRT-PCR. Quantitation was performed using the comparative CT method with *18S* as the endogenous control gene and untreated cells as the calibrator. Bars represent mean fold-change in expression compared to untreated cells. Values are reported as mean ± SD from at least three individual experiments with four biological parallels (one-way ANOVA ****p ≤ 0.0001)

FVII:Ag in the conditioned medium and cell lysates from untreated cells expressing rFVIIwt or rFVII-160R was measured by ELISA. The levels of rFVII-160R in the conditioned medium and cell lysates was significantly reduced compared to rFVIIwt (Fig. 1a). The ratio between secreted FVII (conditioned medium) and intracellular FVII (cell lysates) in untreated cells was calculated and the ratio between the secreted and intracellular rFVII-160R was significantly reduced compared to rFVIIwt (Fig. 1b).

Subsequently, FVII:Ag was measured in conditioned medium and cell lysates from cells treated with the various chemical chaperones. Before treatment, low, but detectable levels of rFVII-160R could be measured in the conditioned medium. No FVII:Ag could be detected in cell lysates or conditioned medium from empty vector cells. Treatment with VX-809, betaine, TMAO, TUDCA or taurine did not affect the FVII:Ag levels (data not shown). However, treatment with 4-PBA increased the levels of rFVII-160R in the conditioned medium up to fourfold and in the cell lysates up to twofold compared to untreated cells (Additional file 1: Fig. S1). Since we observed that treatment with 4-PBA also increased the FVII:Ag levels in the cell lysates, a ratio of secreted (conditioned medium) and intracellular (lysates) FVII was calculated (Fig. 1c, d). The secreted/intracellular ratio of rFVII-160R in cells treated with 4-PBA was increased up to ~ 2.5-fold compared to untreated cells (Fig. 1c). Additionally, the secreted/intracellular ratio of rFVIIwt was increased up to ~ threefold compared to untreated cells (Fig. 1d).

To determine whether the 4-PBA mediated differences in intracellular levels of FVII protein were influenced by increased transcription of the *F7* gene, the mRNA levels were assessed by qRT-PCR. In cells expressing rFVII-160R or rFVIIwt, the FVII mRNA levels were increased up to ~ sevenfold and 15-fold, respectively (Fig. 1e, f). Since 4-PBA is a histone deacetylase inhibitor we examined whether treatment with 4-PBA caused differences in the global acetylation pattern of histone H3. Western blot analysis revealed significantly increased levels of AcH3 after treatment with 10 mM 4-PBA in cells expressing rFVII-160R or rFVIIwt (Additional file 1: Fig. S2).

To exclude the possibility that increased FVII:Ag levels in the conditioned medium was due to reduced degradation of secreted FVII, medium from rFVIIwt cells was incubated ± 10 mM 4-PBA and FVII:Ag levels were subsequently measured at different time points. There were no differences in the FVII:Ag levels after 6, 16, 24 or 48 h of incubation with 4-PBA compared to untreated controls (Additional file 1: Fig. S3). Trypan blue exclusion test showed no differences in viability between untreated cells and cells treated with 4-PBA (data not shown).

### Treatment with 4-PBA slightly enhances the biological activity of rFVII-160R

**Fig. 2:**
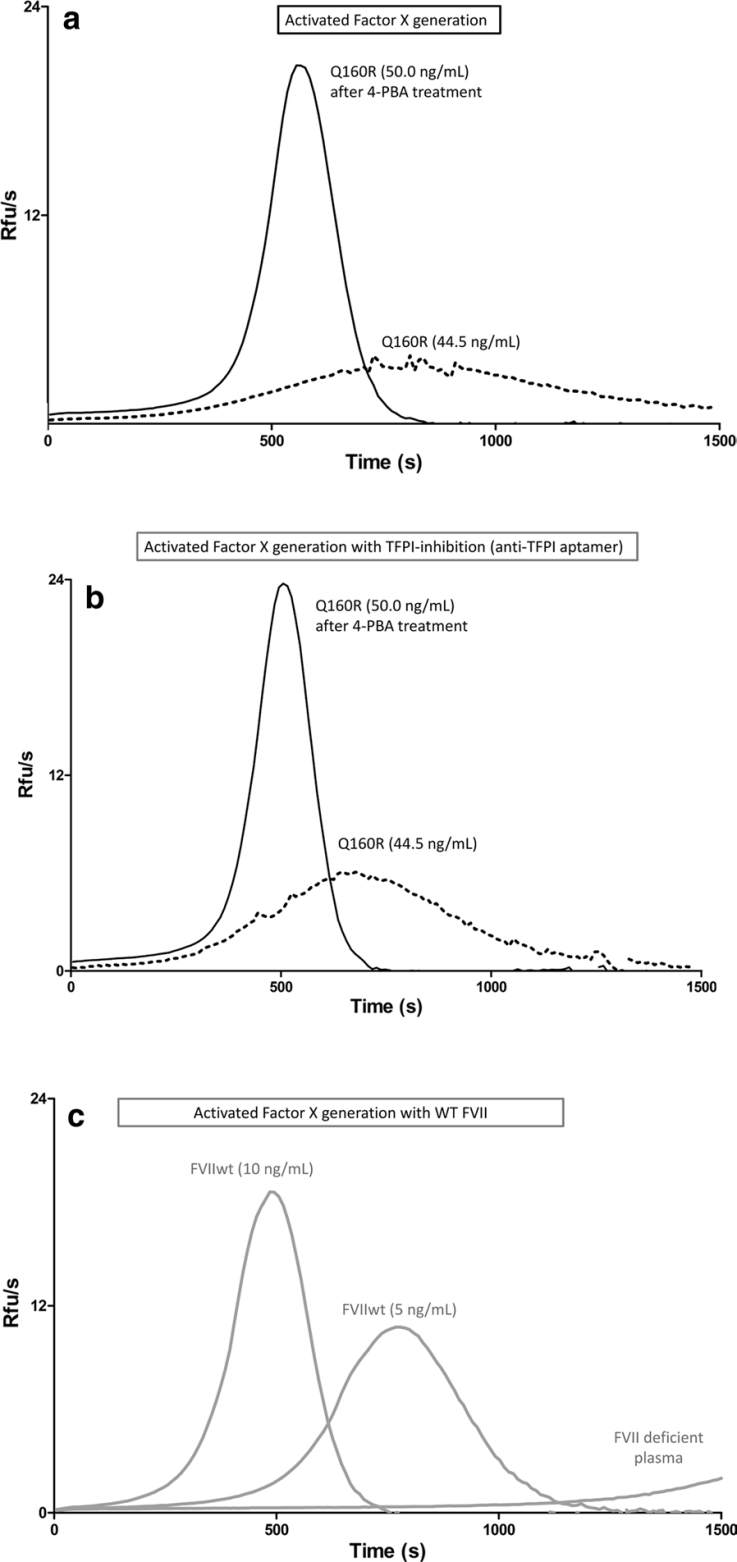
Activity measurements on the rFVII-160R variant. FXa generation activity (Relative Fluorescence Units, Rfu/seconds) in FVII deficient plasma supplemented with conditioned medium. **a** Dotted line, untreated, FVII:Ag 44.5 ng/ml; continuous line, 4-PBA treated, FVII:Ag 50 ng/ml). **b** Medium of the recombinant variant as in A, with inhibition of TFPI by a specific aptamer. **c** FVII deficient plasma supplemented with diluted rFVIIwt medium (FVII:Ag 10 ng/ml; FVII:Ag 5 ng/ml) or with conditioned medium from cells expressing the vector without FVII cDNA. The FXaG shown are representative of 3 independent experiments; all curves have been produced in duplicates, and are reported in three aligned panels for the sake of clarity

The generation of activated FX (FXaG), measured by a fluorogenic substrate specific for the activated enzyme (FXa), was used to evaluate the residual activity of the rFVII-160R. The activity of the secreted variant was measured in FVII deficient plasma supplemented with conditioned medium from cells either untreated or treated with 10 mM 4-PBA, and compared with rFVIIwt in medium. To exclude the possibility that 4-PBA might influence the test, we examined in advance whether 4-PBA in itself affected the FXaG assay. By adding 10 mM 4-PBA (final concentration, 1 mM 4-PBA), the FXaG curves virtually overlapped (Additional file 1: Fig. S4). A high TF concentration was used to magnify the modest residual activity of the recombinant FVII variant, which also makes negligible intrinsic (TF-independent) activation of coagulation. Upon treatment, FXaG was clearly measurable in medium from cells expressing rFVII-160R (Fig. 2a, continuous line) whereas FXaG in medium from cells not treated with 4-PBA was barely detectable (Fig. 2a, dotted line). We specifically magnified the FXaG by inhibiting the main extrinsic pathway inhibitor, TFPI, through an anti-TFPI aptamer. Inhibition of TFPI potentiated FXaG activity of the 4-PBA-induced FVII variant (Fig. 2b, continuous line). Modest effects were evaluated in FXaG performed in conditioned medium from untreated cells (Fig. 2b, dotted line). As positive control (Fig. 2c) we used the rFVIIwt expressed in parallel and appropriately diluted to 10 ng/ml and 5 ng/ml to favour comparison with rFVII-160R. As background (Fig. 2c), we evaluated the FXaG in the FVII deficient plasma added with conditioned medium from cells expressing the vector without FVII cDNA. The background FXaG resulted very low even at 1300 s.

The FXaG activity was compared with the FVII protein concentration (FVII:Ag) in media, which resulted, after the concentration procedure, in 50 ng/ml for the 4-PBA induced variant (Fig. 2a, b). For the rFVIIwt, appropriately diluted to 10 ng/ml and 5 ng/ml, respectively (Fig. 2c). These data clearly indicated that the specific activity of 4-PBA-induced rFVII-160R was much lower than that of rFVIIwt. FXaG parameters were measured in independent preparations of conditioned medium to confirm and evaluate the residual activity of the variant in relation to rFVIIwt, and when exposed to the anti-TFPI aptamer (Table 1). We estimated that the residual activity of 4-PBA-induced rFVII-160R is comprised between 0.16 and 0.33% of rFVIIwt. As compared with rFVIIwt, variant peak heights were slightly higher than expected from time parameters.

**Table 1:** Activated FX generation (FXaG) parameters of the the 4-PBA treated rFVII-160R variant and of rFVIIwt, with (+) and without TFPI inhibition

Anti-TFPI aptamer	rFVII-160R 50 ng/ml		rFVIIwt 10 ng/ml		rFVIIwt 5 ng/ml	
		+		+^a^		+^a^
Lag time (s)	348 ± 97	261 ± 64	205.3 ± 0.1	176	419.4 ± 9.5	354
TTP (s)	719 ± 96	605 ± 51	485.0 ± 0.1	445	778.1 ± 14.4	687
Peak (Rfu/s)	15.0 ± 3.0	19.8 ± 2.0	18.3 ± 0.3	23.0	10.8 ± 0.1	15.0
AUC (Rfu)	3127 ± 172	3496 ± 111	3388 ± 110	3694	3058 ± 37	3401

*TTP* time to peak, *RFU* relative fluorescence units, *AUC* area under the curve

^a^Values reported for rFVIIwt with anti-TFPI addition have been obtained in a single experiment

### Treatment with 4-PBA decreases ER stress

**Fig. 3:**
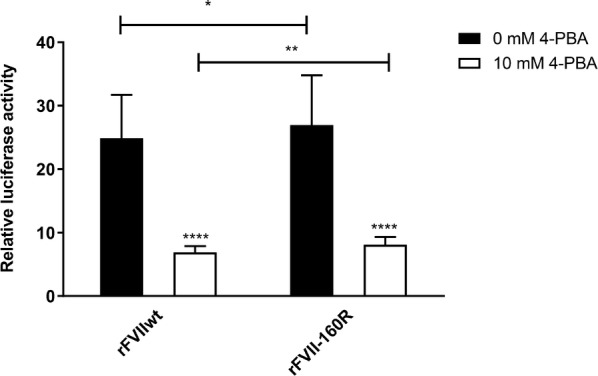
Effect of 4-PBA on ER stress. Cells were transiently co-transfected with the Cignal ERSE reporter construct or the negative control construct and empty vector, pcDNA3-FVIIwt or pcDNA3-p.Q160R. The cells were then treated with 10 mM 4-PBA for 48 h. The ratios between firefly and Renilla luciferase were used for the calculation. The results have been corrected for background activity represented by the negative control. Fold change in luciferase activity was calculated relative to empty vector. Values are reported as mean ± SD fold-change of target gene expression in treated compared to untreated cells from three independent experiments with six biological parallels (unpaired t-test ****p ≤ 0.0001, **p ≤ 0.01, *p ≤ 0.05)

To explore the effect of 4-PBA treatment on ER stress the Cignal ERSE reporter (luciferase) system was employed. In untreated cells, the ER stress levels were significantly higher in cells expressing rFVII-160R compared to rFVIIwt (Fig. 3, black bars), in line with our previous findings [12]. Upon treatment with 10 mM 4-PBA, a significant decrease in luciferase activity was observed in cells expressing rFVIIwt or rFVII-160R compared to untreated cells (Fig. 3, white bars).

### Treatment with 4-PBA corrects the intracellular trafficking of rFVII-160R

**Fig. 4:**
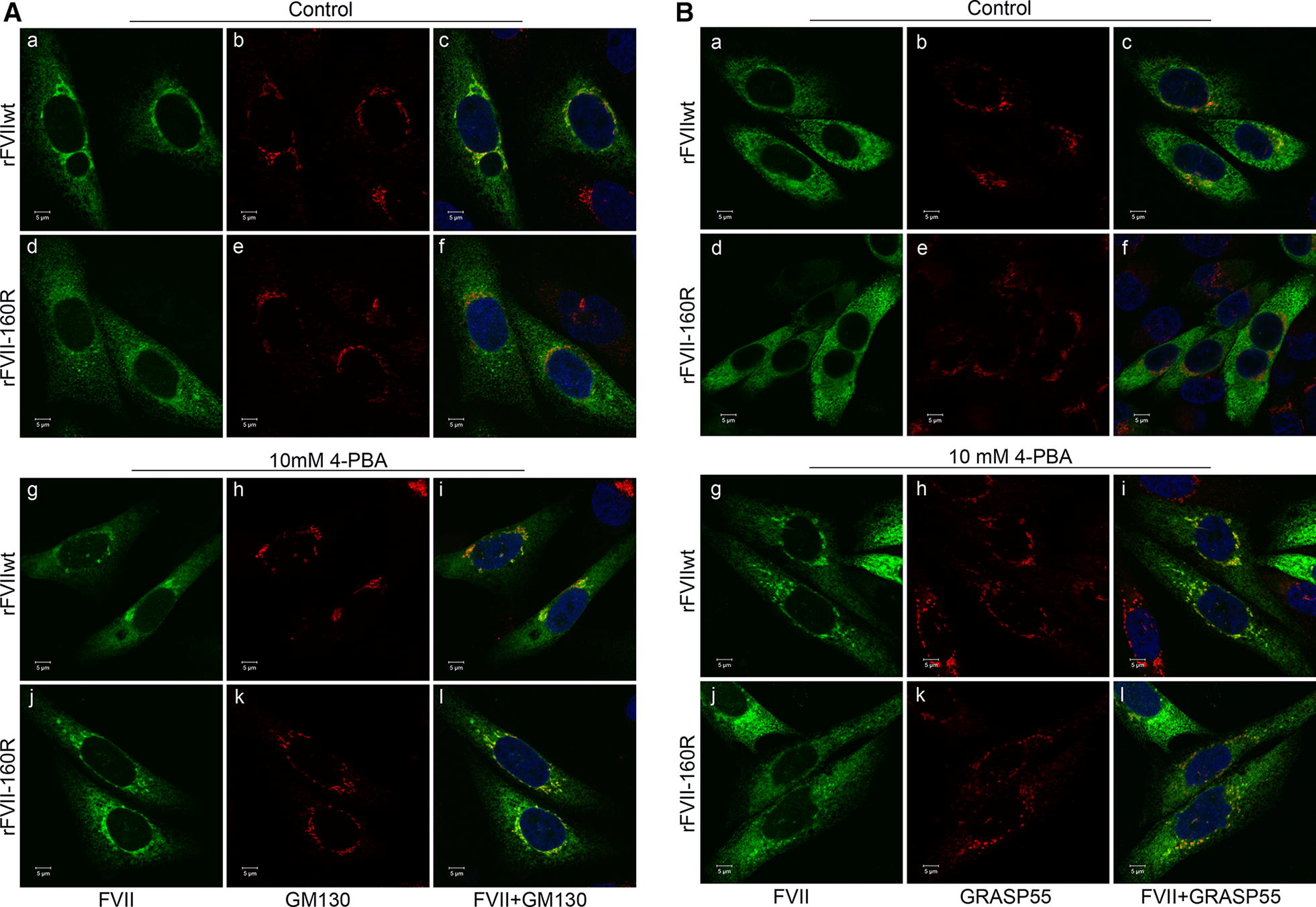
Intracellular localization of rFVII-160R after treatment with 4-PBA. Cells transiently expressing rFVIIwt or rFVII-160R were treated with 4-PBA for 42 h. **A** Confocal images from control cells or cells treated with 10 mM 4-PBA stained with FVII (green, **a**, **d**, **g**, **j**) and GM130 (red, **b**, **e**,** h**, **k**). Co-localized green and red pixels are shown in yellow color (**c**, **f**, **i**, **l**). **B** Confocal images from control cells or cells treated with 10 mM 4-PBA stained with FVII (green, **a**, **d**, **g**, **j**) and GRASP55 (red, **b**, **e**, **h**, **k**). Co-localized green and red pixels are shown in yellow color (**c**, **f**, **i**,** l**). Three independent experiments were performed. Bar 5 µm

Since treatment with 4-PBA improved the secretion of rFVII-160R, we next investigated whether 4-PBA could restore the normal intracellular trafficking of this variant. Thus, we assessed the intracellular localization of rFVIIwt and rFVII-160R in control cells or cells treated with 10 mM 4-PBA by confocal immunofluorescence microscopy using double staining for FVII and various organelle markers. We found that in control cells and cells treated with 4-PBA, both rFVIIwt and rFVII-160R co-localized with the ER marker protein disulfide isomerase (PDI) (Additional file 1: Fig. S5). Moreover, rFVIIwt also clearly co-localized with the Golgi marker GM130 (Manders’ colocalization coefficient 0.21 ± 0.03) (Fig. 4A, c), whereas the co-localization coefficient was only 0.08 ± 0.02 for rFVII-160R and GM130 (Fig. 4A, f). After treatment with 4-PBA, co-localization with GM130 of both rFVIIwt (Manders’ colocalization coefficient 0.20 ± 0.03) (Fig. 4A, i) and rFVII-160R (Manders’ colocalization coefficient 0.21 ± 0.01) (Fig. 4A, l), was detected. We then investigated other possible pathways that might be implicated in the secretion of FVII using double staining of FVII with GRASP55, COPII, Rab-8, Rab-11, ERGIC-53 or syntaxin 8. In control cells, rFVIIwt (Fig. 4B, c), but not rFVII-160R (Fig. 4B, f) co-localized with GRASP55. However, in cells treated with 4-PBA, both rFVIIwt (Fig. 4B, i) and rFVII-160R (Fig. 4B, l) co-localized with GRASP55. Co-localization with GRASP55 was confirmed by superresolution microscopy (Additional file 1: Fig. S6). No co-localization of FVII with Rab-8, Rab-11, ERGIC-53 or syntaxin 8 was observed in either untreated cells or cells treated with 4-PBA (data not shown). However, some degree of co-localization of rFVIIwt and rFVII-160R with COPII could be observed in untreated cells and a slight increase in co-localization of rFVIIwt and rFVII-160R with COPII was observed after treatment with 4-PBA (data not shown). No staining was observed for the negative control using only the secondary antibodies (data not shown). The double negative controls using one of the primary antibodies (FVII or GRASP55) and the two secondary antibodies (donkey anti-goat and donkey anti-mouse) did not show any cross reactivity (data not shown).

## Discussion

Misfolded protein variants are valuable models to investigate whether chemical chaperones can affect and prevent the formation of misfolded structures and reverse the intracellular retention [14]. We have recently reported intracellular retention and activation of ER stress in cells expressing rFVII-160R, possibly as a result of protein misfolding [12]. Based on these findings, we investigated the impact of chemical chaperones on this secretion-defective FVII variant. In addition, the enzymatic activity of FVIIa enabled us to explore whether treatment with chemical chaperones could partially restore the biological function of this variant.

For mammalian overexpression of rFVII, we chose CHO-K1 cells, which are known to produce biologically active FVII with similar post-translational modifications to that of human plasma FVII [18–20]. In accordance with previously reported data [11, 12], we found severely reduced secretion of rFVII-160R compared to rFVIIwt in untreated cells. In our preliminary screen of compounds, we found that the short chain fatty acid 4-PBA could increase the secretion of rFVII-160R to the conditioned medium from the cells. Indeed, the short-chain fatty acid 4-PBA is classified as a chemical chaperone with effects on various misfolded proteins [21]. Increased levels of FVII:Ag could also be detected in the cell lysates from cells treated with 4-PBA. Since the FVII levels in the cell lysates were also increased due to increased FVII production, we calculated a ratio of secreted versus intracellular FVII to clarify the effect on secretion. *F7* mRNA levels were also increased in cells treated with 4-PBA. 4-PBA is known to inhibit the activity of histone deacetylases (HDACs), and thereby induce hyperacetylation of histones resulting in increased gene transcription [22, 23]. In line with this, we observed increased acetylation of histone H3 in response to treatment. The increased mRNA and intracellular protein levels could therefore be a result of increased *F7* gene transcription. However since we observed that the ratio of secreted/intracellular FVII:Ag increased in response to treatment, this suggests that 4-PBA not only affected the synthesis of the FVII protein but also the secretion. We also observed an increased secretion of rFVIIwt upon treatment with 4-PBA. Since overexpression of recombinant proteins may cause a disturbance in the balance of ER protein load and the folding capacity of the host cell [24], we may hypothesize that some rFVIIwt could be temporarily misfolded, which might be corrected by 4-PBA.

4-PBA is a FDA-approved drug originally designed for the treatment of urea cycle disorders, but is reported to act as a chemical chaperone [25]. It was also tested in a short-term clinical trial as a therapeutic approach for cystic fibrosis in patients harbouring the ΔF508 variant of the cystic fibrosis transmembrane conductance regulator [26]. Interestingly, 4-PBA has been reported to increase the secretion of other coagulation factor variants such as FVIII-F309S [27], FIX-R294Q [17], and protein C-A267T [16]. Previously, it was demonstrated that 4-PBA could rescue various mutant proteins arrested in the ER allowing them to be expressed on the cell surface and to be functionally correct [28, 29]. Before treatment, FXaG was barely detectable in conditioned medium from cells expressing rFVII-160R. This is in agreement with data published by Kemball-Cook et al. [10], who showed that the activated form of the mutant recombinant protein had reduced affinity for TF and, in the presence of soluble TF, had less than 5% of the ability of rFVIIwt to activate FX. Accordingly, evaluation of the FXa generation parameters before 4-PBA treatment was not feasible because mutant curves were not suitable for quantitative analysis. After treatment with 4-PBA, FXaG was clearly measurable in medium from cells expressing rFVII-160R. The measured residual activity of the 4-PBA-induced rFVII-160R indicated that the secreted variant was functionally active and still regulated by TFPI. On the other hand the specific activity of the 4-PBA-induced rFVII-160R was still considerably reduced compared to rFVIIwt, as expected for FVII molecules bearing important structural changes. Particularly, the replacement of the glutamine side chain by the larger, positively charged arginine side chain in FVII-160R would disrupt hydrogen bonding networks and destabilize protein inter-domain areas [10]. It is tempting to speculate that folding disturbances in the EGF-2 protease region of FVII-160R, probably responsible both for barely detectable affinity for TF and for the secretion defect, would be partially overcome by the 4-PBA. The chaperone effects on secretion were better than those on the activity, which could be interpreted in light of the arduous recovery of the very specific protease FVIIa activity, highly potentiated by the cofactor TF binding, impaired by the Q160R change.

We have recently reported activation of ER stress in cells expressing rFVII-160R [12]. In line with previous reported data [30] we found that 4-PBA could alleviate ER stress in our cell model. We might therefore hypothesize that the reduced ER stress and the slight increase in biological activity of rFVII-160R could be a result of enhanced folding of the FVII mutant protein, as has been reported for other misfolded protein variants [31]. However, a limitation to this study is that due to the lack of access to primary hepatocytes from patients harbouring the p.Q160R variant, we are relying on a cell model based on overexpression. Therefore, with this model, it might be difficult to assess whether the help conferred by chemical chaperones could be sufficient to obtain therapeutic significance for severe mutations in sophisticated enzymes like coagulation serine proteases. It was previously demonstrated that the secretion of newly synthesized FVIII, which was misfolded in the ER and caused UPR activation and oxidative stress, could be improved by antioxidant treatment [32]. However, we did not find any effect on FVII secretion by the treatment with the antioxidants betaine or taurine. Nevertheless, further studies would be necessary to clarify whether misfolded rFVII-160R could cause oxidative stress and whether treatment with other antioxidants might have an effect on its secretion.

4-PBA is reported to improve mislocalization of proteins associated with human diseases [33]. Previous in vitro studies on rFVII-160R demonstrated a defective intracellular transport with ER retention [11]. This is in line with our observations, demonstrating only a slight co-localization of rFVII-160R with the Golgi marker in untreated cells. However, upon treatment with 4-PBA, a clear co-localization of rFVII-160R with the Golgi marker was observed, suggesting that the variant was transported to the Golgi in response to the treatment. Interestingly, we did observe a slight increase in co-localization of rFVII-160R with COPII after treatment with 4-PBA. This could be expected since COPII is involved in anterograde transport of cargo proteins from the ER to the Golgi [34]. However, the small increase in co-localization does not allow us to make any strong conclusions about the transport of rFVII-160R via this pathway upon 4-PBA treatment. We also found rFVII-160R to be localized to GRASP55-positive structures upon treatment with 4-PBA. GRASPs were originally identified as factors required for the stacking of Golgi cisternae [35, 36]. However, they were demonstrated to be involved in the unconventional secretion of proteins that bypasses the Golgi, such as the ΔF508-CFTR mutant [37]. Correspondingly, we previously reported increased secretion of PC-A267T via the GRASP pathway upon treatment with 4-PBA [16].

## Conclusions

In conclusion, we report here for the first time that a chemical chaperone could improve the secretion and restore the intracellular trafficking of a missense FVII variant associated with congenital FVII deficiency. Further studies using human pluripotent stem cell derived hepatocytes expressing this FVII variant are in progress in order to assess the relevance of this therapy in physiological FVII-producing cells.

## Methods

### Nomenclature of gene variants

The gene variant p.Q160R was named according to the official HGVS recommendation [38] and is reported in the FVII gene variant database [8].

### Chaperones and antibodies

The chemical chaperones 4-PBA, taurine, betaine, trimethylamine *N*-oxide (TMAO) and tauroursodeoxycholic acid (TUDCA) were purchased from Sigma Aldrich (Saint Louis, MO, USA). VX-809 (a pharmacological chaperone) was purchased from Selleckchem (Houston, TX, USA). Commercial antibodies are listed in Additional file 1: Table S1.

### Cell cultures

The Chinese hamster ovary cell line CHO-K1 (no endogenous FVII expression) was provided by the American Type Culture Collection (ATCC, Rockville, MD, USA, CCL-61). The cells were maintained as previously described [12].

### Expression vectors and transfection

pcDNA™3.1^(+)^ (pcDNA3) was used as empty control vector (Thermo Fisher Scientific, Rockford, IL, USA). pcDNA3-FVII wild-type (FVIIwt) and pcDNA3-p.Q160R were generated as previously described [12]. For transient and stable transfection, CHO-K1 cells were transfected with cDNA constructs using Lipofectamine LTX (Thermo Fisher Scientific) according to the manufacturer’s instructions. Stable cell lines were generated as previously described [12] and maintained in the presence of 400 μg/ml G-418.

### Treatment with chemical chaperones

Cells with stable expression of rFVIIwt or rFVII-160R were seeded onto 6-well or 24-well CellBIND plates (Corning Incorporated, Kennebunk, ME, USA) and treated with various concentrations of chemical chaperones diluted in DMEM supplemented with 1% FBS and 10 μg/ml vitamin K1. Treatment with VX-809, TMAO, TUDCA or taurine was conducted for 24 h. 4-PBA and betaine treatment was performed for 48 h and 72 h, respectively. To test for possible toxic effects of 4-PBA, the cell viability was measured by trypan blue exclusion assay.

### FVII antigen determination

Conditioned medium was collected and the cells were lysed as previously described [12]. ELISA was performed on conditioned medium and lysates using the VisuLize™ Factor VII Antigen ELISA Kit (Affinity Biologicals, Ancaster, ON, Canada) according to the manufacturer’s protocol. Colorimetric output was measured using a SpectraMax Plus spectrophotometer (Molecular Devices, Sunnyvale, CA, USA).

### Generation of activated FX in medium

Cells with stable expression of rFVIIwt or the rFVII mutants were seeded onto 15 cm dishes (Thermo Scientific) and treated with ± 10 mM 4-PBA. Conditioned medium was harvested and concentrated approximately 30 times through the Amicon Ultra centrifugal filter devices (cut-off 10 kDa, Millipore, Carrigtwohill, Country Cork, Ireland). Generation of activated FX (FXaG) was performed in FVII deficient plasma (George King, Bio-Medical Inc., Overland Park, KS, USA) added simultaneously with the concentrated medium. The assay was performed as previously described [39] using 50 pM TF (Innovin, Dade Behring, Marburg, Germany) to initiate FXaG. FXaG was also evaluated by inhibition of the TF pathway inhibitor (TFPI) through 1 μM anti-TFPI RNA aptamer [40]. All samples were tested in duplicates in at least three independent experiments. Parameters of FXaG (lag time, peak, time to peak and area under the curve) were obtained using GraphPad Prism 5.0 software (GraphPad Software, La Jolla, CA, USA).

### Western blot analysis

Equal amounts of protein from cell lysates of cells treated with 4-PBA were separated by SDS-PAGE with Mini-PROTEAN^®^ TGX AnyKD precast gels (Bio-Rad, Hercules, CA, USA) as previously described [12]. The membranes were incubated overnight at 4 °C with primary antibodies as indicated in the figure legends. The membranes incubated with the appropriate secondary antibodies (Cell Signaling Technology, Danvers, MA, USA or Santa Cruz Biotechnology, Dallas, TX, USA) and immune complexes were visualized as previously described [12].

### Cignal ERSE reporter assay

Cignal reporter assay was performed with the Cignal ER-stress response element (ERSE) (luciferase) kit (SABiosciences, Frederick, MD, USA) as previously described [12]. Each sample was normalized by dividing the test reporter activity by the negative control reporter. The results are presented relative to the empty vector.

### Quantitative RT-PCR (qRT-PCR)

Total RNA was isolated and cDNA was synthesised as previously described [12]. To analyse *F7* mRNA levels, qRT-PCR was performed using the TaqMan Gene Expression Master Mix on a 7900HT Fast Real-Time PCR system (Thermo Fisher Scientific) and the following Taqman assays: Hs01551992_m1 (*F7*) and Hs99999901_s1 (*18S*) (both from Thermo Fisher Scientific).

### Confocal immunofluorescence microscopy

Cells were seeded on 4-well Lab-Tek II chamber slides (Thermo Fisher Scientific) and transfected with the respective FVII cDNA constructs as described above. The cells were then treated with ± 10 mM 4-PBA as described above. Immunostaining was performed 42 h after treatment with 4-PBA using antibodies against FVII the ER marker protein disulfide isomerase (PDI), the Golgi marker GM130, GRASP55, Ras-related protein (Rab)-11, Rab-8, coat protein (COP)II, ER-Golgi intermediate compartment (ERGIC)-53 or syntaxin 8. Alexa Fluor^®^ 488 donkey anti-goat, Alexa Fluor^®^ 568 donkey anti-mouse or Alexa Fluor^®^ 568 donkey anti-rabbit (all from Thermo Fisher Scientific) were used as secondary antibodies. Cells were mounted in the SlowFade^®^ Gold Antifade reagent with DAPI (Thermo Fisher Scientific) and examined with a Zeiss LSM 710 confocal microscope (Carl Zeiss MicroImaging GmbH, Jena, Germany) and a Zeiss LSM 880 Airyscan (Zeiss). Image processing was performed with basic software ZEN 2011 and ZEN 2 blue (Zeiss). Negative controls with only secondary antibodies as well as double negative controls using one of the primary antibodies and both secondary antibodies were included. The Manders’ co-localization coefficient was calculated with the ZEN 2011 software as described before [41].

### Airyscan imaging

The cells were examined with a Zeiss LSM 880 Airyscan microscope (Carl Zeiss MicroImaging GmbH, Jena, Germany), equipped with an Ar-Laser Multiline (458/488/514 nm), a DPSS-561 10 (561 nm), a Laser diode 405-30 CW (405 nm), and a HeNe-laser (633 nm). The objective used was a Zeiss plan-Apochromat 63xNA/1.4 oil DICII. The images were acquired with the Airyscan detector with a voxel size of 0.035 × 0.035 × 0.144 µm for high resolution imaging and super-resolution processing resulting in a final resolution of 120 × 120 × 350 nm. Image acquisition and processing were done with the ZEN 2.3 SP1 basic software (Carl Zeiss), or with ZEN 2.3 Blue (Carl Zeiss).

### Statistical analysis

The statistical analyses were performed using GraphPad Prism 7.0. Comparisons were performed using one-way ANOVA or unpaired t-test. A p-value of ≤ 0.05 was considered as statistically significant.

## Supplementary information

**Additional file 1: Table S1.** List of commercial antibodies used in Western blot analysis and immunofluorescence. **Fig. S1.** Expression levels of rFVII-160R in cells treated with 4-PBA. **Fig. S2.** Acetylation of histone H3 in in cells expressing rFVIIwt and rFVII-160R. **Fig. S3.** Treatment with 4-PBA does not affect FVII:Ag stability in conditioned medium. **Fig. S4.** No effect of 4-PBA in itself on FXaG. **Fig. S5.** Confocal images of cells expressing expressing rFVIIwt and rFVII-160R treated with 4-PBA. **Fig. S6.** Airyscan images of cells expressing rFVIIwt and rFVII-160R treated with 4-PBA.

## Data Availability

Not applicable.
