# Supplementary material for: The effect of the chemical chaperone 4-phenylbutyrate on secretion and activity of the p.Q160R missense variant of coagulation factor FVII
Source: Cell Biosci. 2019 Aug 27;9:69. doi: 10.1186/s13578-019-0333-8 (PMC6712599; doi:10.1186/s13578-019-0333-8)
Supplement: Supplementary file 1 — Additional file 1: Table S1. List of commercial antibodies used in Western blot analysis and immunofluorescence. Fig. S1. Expression levels of rFVII-160R in cells treated with 4-PBA. Fig. S2. Acetylation of histone H3 in in cells expressing rFVIIwt and rFVII-160R. Fig. S3. Treatment with 4-PBA does not affect FVII:Ag stability in conditioned medium. Fig. S4. No effect of 4-PBA in itself on FXaG. Fig. S5. Confocal images of cells expressing expressing rFVIIwt and rFVII-160R treated with 4-PBA. Fig. S6. Airyscan images of cells expressing rFVIIwt and rFVII-160R treated with 4-PBA. [file 13578_2019_333_MOESM1_ESM.docx]

| **Table S1.** List of commercial antibodies used in Western blot analysis and immunofluorescence | |
| --- | --- |
| **Antibody name** | **Company** |
| AcH3 (Lys 9) | Cell Signaling Technology, Danvers, MA, USA |
| β-actin | Cell Signaling Technology, Danvers, MA, USA |
| FVII | R&D Systems, Minneapolis, MN, USA |
| PDI | Enzo Life Sciences, Farmingdale, NY, USA |
| GM130 | BD Biosciences, San Jose, CA, USA |
| GRASP55 | Santa Cruz Biotechnology, Dallas, TX, USA |
| Rab-11 | BD Biosciences, San Jose, CA, USA |
| Rab-8 | LifeSpan BioSciences, Seattle, WA, USA |
| COPII | Thermo Fisher Scientific, Rockford, IL, USA |
| ERGIC-53 | Santa Cruz Biotechnology, Dallas, TX, USA |
| Syntaxin 8 | BD Biosciences, San Jose, CA, USA |

**Fig. S1. Expression levels of rFVII-160R in cells treated with 4-PBA.** Cells with stable expression of rFVII-160R were treated with 4-PBA for 48 h. FVII:Ag levels in conditioned medium (black bars) or cell lysates (white bars) were examined by ELISA. Values are expressed relative to non-treated cells and represent mean ± SD of at least three individual experiments performed in duplicates (***p≤0.001, **p≤0.01, *p≤0.05).

**

**

**Fig. S2. Acetylation of histone H3 in in cells expressing rFVIIwt and rFVII-160R.** AcH3 and β-actin was assessed by Western blot analysis in lysates from CHO-K1 with stable expression of empty vector (pCDNA3) or rFVIIwt treated with 10mM 4-PBA for 48 h. (A) One representative experiment is shown. (B) Fold increase of AcH3 are shown as bars representing mean ±SD of two individual experiments (N=2). Bands representing AcH3 were quantified and normalized to β-actin. The results are presented relative to untreated cells (0 mM 4-PBA).

**

**

**Fig. S3. Treatment with 4-PBA does not affect FVII:Ag stability in conditioned medium.** Conditioned medium from CHO-K1 with stable expression of rFVIIwt was removed and treated with 0mM (circles) or 10mM (squares) 4-PBA for 0, 6, 16, 24 and 48 h. FVII:Ag levels in conditioned medium were examined by ELISA. Values are expressed relative to 0 h and represent mean from one experiment performed in duplicates.

**

**

**Fig. S4. No effect of 4-PBA in itself on FXaG.**

FXaG in diluted (1:20) pooled normal plasma with high tissue factor concentration (50 pM) and 1 mM 4-PBA. Gray points/lines, 4-PBA addition; black points/lines, no 4-PBA added. Left panels, fluorescence (Relative Fluorescence Units, Rfu) over time. Right panels, first derivative of fluorescence. All curves have been produced in a single experiment, and represent the mean of 3 simultaneous evaluations.

**
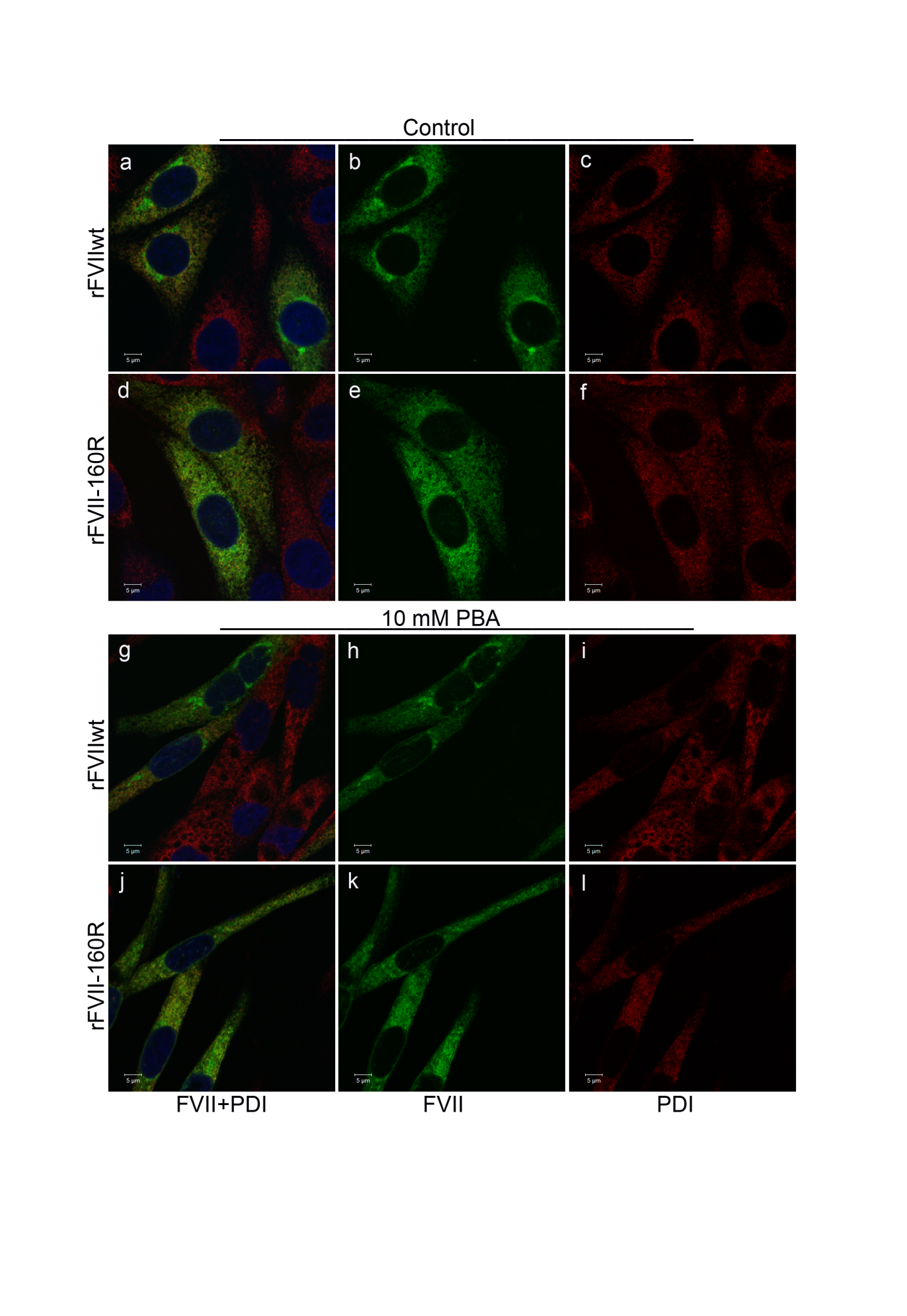
**

**Fig. S5. Confocal images of cells expressing expressing rFVIIwt and rFVII-160R treated with 4-PBA.** Cells with transient expression of rFVIIwt or rFVII-160R were treated with 4-PBA for 42 h. Confocal Airyscan images from cells stained with FVII (green, b, e, h, k) and PDI (red, c, f, I, l). Co-localized green and red pixels are shown in yellow color (a, d, g, j). Bar 5 µm

**
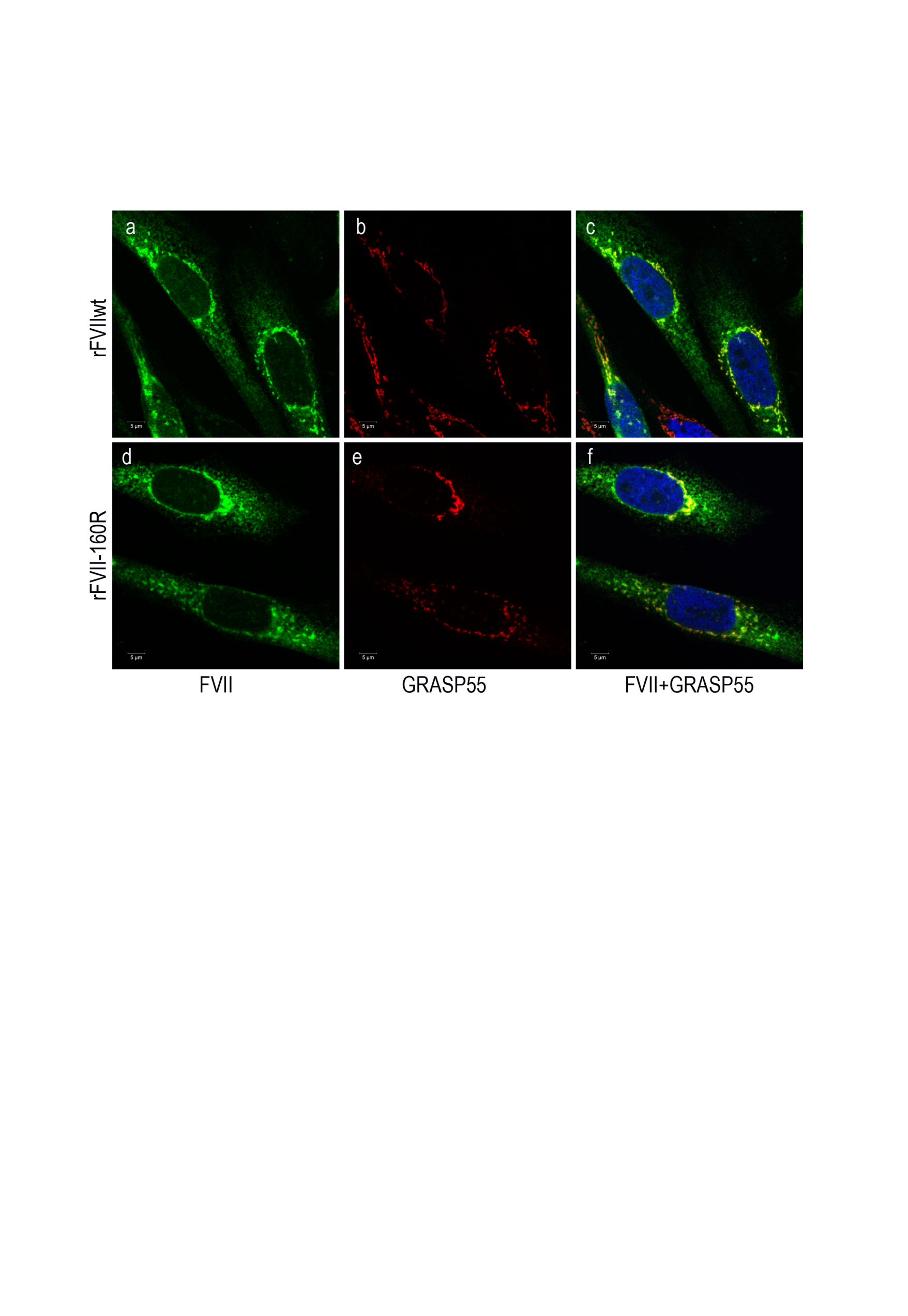
**

**Fig. S6. Airyscan images of cells expressing expressing rFVIIwt and rFVII-160R treated with 4-PBA.** Cells with transient expression of rFVIIwt or rFVII-160R were treated with 4-PBA for 42 h. Confocal Airyscan images from cells stained with FVII (green, a, d) and GRASP55 (red, b, e). Co-localized green and red pixels are shown in yellow color (c, f). Bar 5 µm
